# Beyond risk: rethinking hospitalization for suicidal individuals

**DOI:** 10.3389/fpsyt.2025.1684927

**Published:** 2025-09-04

**Authors:** Christian Greiner, Jacqueline Huber, Paco Prada, Matthew Large

**Affiliations:** ^1^ Liaison Psychiatry and Crisis Intervention, Department of Psychiatry, Geneva University Hospital, Geneva, Switzerland; ^2^ Mental Health, St Vincent’s Hospital Sydney, Darlinghurst, Australia; ^3^ Discipline of Psychiatry and Mental Health, University of New South Wales, Sydney, Australia

**Keywords:** suicide prevention and intervention, risk assessment, psychiatric hospitalization, clinical formulation, relational approach

## Introduction

Efforts to prevent suicide have traditionally focused on identifying “high-risk” groups and protecting them, often through psychiatric hospitalization, which is considered the ultimate safeguard. However, decades of research have demonstrated that predicting individual suicide deaths is impossible at both the point of entry (1) and the point of exit from hospital (2). Suicide risk assessments, once considered foundational to practice, have shown limited reliability and poor predictive value. Meta-analyses consistently reveal that most individuals labeled as “high risk” do not die by suicide, while many who do are not identified (3, 4). Similar concerns arise from reviews of deaths by suicide occurring shortly after assessments in healthcare settings, highlighting that the assessment process itself offers little protection when not paired with sustained and relational care (5). Only recently have these facts started to gain mainstream recognition (6); yet, clinical practice, likely influenced by community expectations, continues to be shaped by the entrenched belief that admission and discharge decisions hinge on the illusion of precise risk assessment. In fact, research highlights how clinicians’ emotional regulation abilities may affect risk evaluations and hospitalization decisions, especially in uncertain and emotionally charged clinical interactions (7).

Additionally, meta-analytic data indicate that suicide rates remain alarmingly high immediately after discharge, even when the protective effect of hospitalization is assumed to peak (2, 8, 9), concluding that there is no evidence that hospitalization reduces suicide risk (10). In fact, there exists recent evidence that hospitalization may increase risk of suicide attempts, though those who attempted suicide within the past day were found to possibly benefit from hospitalization (11). As a result, we are now facing a significant ethical and clinical reckoning. If accurate risk assessment does not guide decision-making, then what does? If admission does not reliably mitigate the risk of suicide, whom should we admit, and what should admission aim to achieve?

In this piece, we argue that hospitalization based on “risk assessment” is inappropriate, unfeasible, and potentially harmful. Instead, we should focus on goals that do not aim to reduce “risk”, such as pursuing highly effective relational approaches for individuals presenting to hospitals with suicidality.

## The limits of suicide prediction

It is now widely recognized that our ability to predict suicide remains effectively “no better than chance” (3), with risk instruments inevitably producing numerous false positives and negatives. Even if we were to consider stratified risk assessment as beneficial, all individuals admitted to hospitals for psychiatric care fall into the “high-risk” category, ultimately rendering this categorization ineffective. Moreover, widely used instruments such as the Columbia-Suicide Severity Rating Scale (C-SSRS) have demonstrated poor predictive value in emergency settings, with large U.S. and Swedish cohorts reporting negligible discrimination and very low positive predictive values, and additional findings suggesting that ideation ratings were insensitive to risk after discharge (12–14). Furthermore, an excessive focus on risk categorization may hinder care. As Smith et al. described, an “ appropriately narrow focus” on diagnosis and risk factors can lead to hasty or inconsistent decision making, compromising therapeutic engagement (15). For this reason, and given the ineffectiveness of suicide risk assessments, it is crucial to reassess the role of hospitalization in suicidal individuals.

## Hospitalization and suicide outcomes

Psychiatric admissions are frequently viewed as the ultimate safeguard for patients with suicide. However, evidence indicates that admissions do not appear to prevent suicide. In fact, the period following discharge is exceptionally high-risk, a result that remains unaffected by the duration of an individual’s hospital stay (2). A meta-analysis of global data revealed that the suicide rate in the first three months post-discharge was approximately 100 times the global average. For patients admitted because of suicidal thoughts or attempts, the relative risk was even greater, reaching approximately 200-fold (8).

This post-discharge vulnerability may not necessarily suggest that the act of hospitalization is inherently harmful or ineffective. It is often argued, for example, that these statistics highlight both the severity of the crises that clinicians admit for inpatient care, and the structural weaknesses in managing transitions out of the hospital (16); or that the sudden loss of structure, disconnection from therapeutic relationships, and delays in starting outpatient follow-up may contribute to this period of increased risk (17, 18). It should also be acknowledged that in some specific situations such as acute psychotic episodes where suicidality may be present, hospitalization retains utility by providing medical stabilization and access to pharmacological or structured interventions that are not always feasible in community settings. However, there is also evidence that hospitalization itself may intensify distress and incidence of suicide attempts, particularly when it is perceived as coercive, depersonalizing, or disconnected from the patient’s subjective needs (11, 19, 20), or when individuals are involuntary hospitalized (21). Interestingly, there is evidence that rates of in-patient suicide vary widely, suggesting that differences in hospital practices may contribute to suicide rate heterogeneity (22).

## Heterogeneity and hospitalization

Randomized trials that specifically evaluated hospitalization as an intervention are limited. However, studies on specific clinical interventions have provided valuable insights. For example, brief suicide-focused therapies, safety planning, and post-crisis outreach have all demonstrated benefits. A recent meta-analysis of acute care interventions found that a single targeted session, typically involving a collaboratively developed safety plan and connection to follow-up care, was linked to approximately 30% fewer subsequent suicide attempts (23). In contrast, few randomized controlled trials have directly examined the impact of inpatient admission, with existing studies largely relying on correlational designs (24). While some structured, time-limited therapeutic interventions during hospitalization, such as those focusing on emotion regulation or relational stabilization, have shown promise (25), many routine practices (e.g., constant observation, safety contracts, or seclusion) lack robust empirical support, and interventions and outcome measures are heterogeneous (26, 27). Importantly, none has demonstrated efficacy in suicide prevention.

In practice, hospitalization often functions less as a targeted clinical intervention and more as an institutional reflex aimed at managing acute crises and institutional anxiety (28). This underscores the importance of critically examining the conditions under which it is used, what it aims to achieve, and how it is embedded within a broader continuum of care. If not carefully conceptualized and supported by post-discharge continuity, hospitalization risks being simply an episode of respite, only with the potential for significant harm. To fully grasp the persistent reliance on hospitalization, despite limited evidence, we must turn our attention to the powerful symbolic and emotional expectations it carries for clinicians, patients, and families.

## Admissions are nonetheless likely to occur

Despite the limited empirical support for its effectiveness, hospitalization remains widely regarded as a life-saving intervention. Within the mental health ecosystem, it serves as a symbol of reassurance: clinicians view it as a way to “contain risk,” families see it as an act of protection, and those in crisis perceive it as a safe haven and signifier of the seriousness of their suffering (29, 30). Concerns about litigation, institutional scrutiny, and personal responsibility can lead to decisions that are procedurally safer but not necessarily based on therapeutic rationale (31). Meanwhile, individuals in crises may experience admission as either validation or condemnation, sometimes finding temporary relief, while at other times reinforcing narratives of helplessness (19, 23). Confronted with intolerable uncertainty, high emotional stakes, and institutional expectations, clinicians may resort to hospitalization not out of belief in its therapeutic value but as a response to systemic anxiety and medicolegal pressures (29, 32). Thus, hospitalization becomes a symbolic gesture of having acted and protected rather than enacting appropriate care in response to a clinical formulation.

In the current cultural context, admissions for individuals expressing suicidality frequently occur even in the absence of clear evidence for their effectiveness, and given the marked variability in outcomes across hospitals, such decisions may at times be justifiable. Within this clinical reality, we must ask: how might hospital admission serve a meaningful therapeutic function? We argue that it could be conceptualized not merely as a containment strategy, but as a time-limited, formulation-based clinical intervention. Preliminary evidence supports the efficacy of brief admissions when combined with targeted suicide-focused interventions (25). Structured psychotherapeutic tools such as safety planning, crisis response planning, and brief mentalization-based or cognitive-interpersonal strategies can be integrated into inpatient care to reduce suicidal ideation and behaviors (26, 33, 34) though, to our knowledge, not deaths by suicide. Rather than relying on standardized risk thresholds, the decision to hospitalize should emerge from an individualized understanding of the patient’s psychological functioning, interpersonal dynamics and current stressors; that is, through a collaborative clinical formulation (24, 35).

## Goals, not risk

Confronted with the limitations of current knowledge and lack of reliable predictive tools, clinicians should shift from an anxiety-driven, risk-focused model to one that prioritizes distress-oriented, relational care. While qualitative studies have explored individuals’ expectations of the inpatient environment, such as sanctuaries, safety, respite, and therapeutic settings (29), there is a lack of research on specific treatment goals. Although some data exists on clinicians’ opinions regarding goals (32), there is little formal comparison between the two perspectives in the literature.

Care goals should be collaboratively established with those seeking care, and some recommendations may include the following:

- Distress management: This might involve implementing structured interventions that target emotional dysregulation, identity confusion, trauma, and epistemic mistrust, especially in individuals with personality vulnerabilities or histories of invalidation. Approaches such as Mentalization-Based Treatment (MBT), brief relational crisis models, and other personalized methods can guide these interactions (34, 36).
- Building and repairing trust: Hospitalization often disrupts continuity of care, taking patients away from familiar environments, routines, and therapeutic relationships. For many, particularly those with a history of relational trauma, this disruption can intensify feelings of mistrust and alienation. Therefore, clinicians could prioritize relational repair by transparently explaining decisions, validating the individual’s subjective experience, and ensuring continuity of care both before and after admission (29, 37).
- Supporting the transition back to community: The period following discharge is one of the most critical and vulnerable phases in a person’s trajectory. However, due to resource limitations, follow-up care is often fragmented, delayed, or misaligned with patient needs. An effective transition requires bridging the gap between inpatient and outpatient care through warm handovers, coordinated plans, and individualized crisis management tools (38). Collaborative crisis planning, which involves engaging patients in identifying triggers, warning signs, and protective strategies, has shown promise in enhancing safety and engagement post-discharge (39).

## Conclusion

Clinicians considering hospitalizing a patient should not understand it as a calculated response to risk, but as a relational and pragmatic intervention for addressing intolerable distress. At the same time, clinicians must remain alert to emerging evidence suggesting that admission may, in some cases, heighten suicide risk. The aim, therefore, cannot be limited to the preservation of life, especially given the lack of evidence that this is reliably achievable, but must extend to relational and systemic supports that help make life livable. This requires broad-scale shifts in training, psychiatric treatment and environmental design, clarification of appropriate outcome measures, a heavy emphasis on clinical research, and change in how suicide is understood in public discourse.
